# PI3K-GLUT4 Signal Pathway Associated with Effects of EX-B3 Electroacupuncture on Hyperglycemia and Insulin Resistance of T2DM Rats

**DOI:** 10.1155/2016/7914387

**Published:** 2016-08-31

**Authors:** Bing-Yan Cao, Rui Li, Huan-Huan Tian, Yan-Jia Ma, Xiao-Gang Hu, Ning Jia, Yue-Ying Wang

**Affiliations:** ^1^Department of Qigong and Tuina, Xiyuan Hospital, China Academy of Chinese Medical Sciences, Beijing, China; ^2^School of Acupuncture, Moxibustion, and Tuina, Beijing University of Chinese Medicine, Beijing 100029, China

## Abstract

*Objectives.* To explore electroacupuncture's (EA's) effects on fasting blood glucose (FBG) and insulin resistance of type 2 diabetic mellitus (T2DM) model rats and give a possible explanation for the effects.* Method.* It takes high fat diet and intraperitoneal injection of streptozotocin (STZ, 30 mg/kg) for model preparation. Model rats were randomly divided into T2DM Model group, EA weiwanxiashu (EX-B3) group, and sham EA group (*n* = 12/group). EA (2 Hz continuous wave, 2 mA, 20 min/day, 6 days/week, 4 weeks) was applied as intervention. FBG, area under curve (AUC) of oral glucose tolerance test (OGTT), insulin resistance index (HOMA-IR), pancreatic B cell function index (HOMA-B), skeletal muscle phosphorylated phosphatidylinositol-3-kinase (PI3K), glucose transporter 4 (GLUT4), and membrane GLUT4 protein expression were measured.* Results.* EA weiwanxiashu (EX-B3) can greatly upregulate model rat's significantly reduced skeletal muscle PI3K (Y607) and membrane GLUT4 protein expression (*P* < 0.01), effectively reducing model rats' FBG and AUC of OGTT (*P* < 0.01). The effects are far superior to sham EA group.* Conclusion.* EA weiwanxiashu (EX-B3) can upregulate skeletal muscle phosphorylated PI3K protein expression, to stimulate membrane translocation of GLUT4 and thereby increase skeletal muscle glucose intake to treat T2DM.

## 1. Introduction

Type 2 diabetes mellitus (T2DM) accounts for nearly 95% of total diabetic patients worldwide [1, 2]. The disease is characterized by hyperglycemia [3] caused by impaired insulin secretion or peripheral insulin resistance [4]. As a progressive chronic disease, it is closely related with complications such as diabetic nephropathy, diabetic foot, and diabetic ophthalmopathy [5]. Besides, it is also one of the leading causes of cardiovascular disease [6]. The prevalence of T2DM among adults is predicted to be more than 3.47 million [7], making mechanism study on the disease of great value.

Glucose transporter 4 (GLUT4) is highly expressed in major tissues responding to insulin including skeletal muscle, liver, and adipose tissue [8]. It actively transports glucose from blood into the cell when translocated to cell membrane [9, 10]. Given that skeletal muscle accounts for more than 75% of insulin-stimulated glucose disposal [11] and is the tissue with highest GLUT4 expression [12], skeletal muscle GLUT4 plays an important role in the pathogenesis of type 2 diabetes [13]. It therefore not only performs as the major mediator of glucose uptake of skeletal muscle but also maintains glucose homeostasis of the whole body [14–16]. It has been reported that overexpression of GLUT4 in skeletal muscle improves glucose homeostasis [17] by lowering blood glucose [16, 18], increasing insulin-stimulated glucose transport [19], and restoring pancreatic B cell morphology [20]. And targeted disruption of skeletal muscle GLUT4 expression will cause severe insulin resistance and glucose intolerance [15]. Researchers have realized that decrease in GLUT4 expression is closely related to insulin resistance [21], and methods that enhance GLUT4 translocation to the membrane or increase its protein expression can relieve insulin resistance [11].

Acupuncture is proved to have certain effect on T2DM and was thereby recommended by World Health Organization (WHO) as possible therapeutic method for the disease [22]. Previous researches proved that electroacupuncture (EA) has certain hypoglycemic effect [23] and can improve glucose tolerance [24, 25]. And it is demonstrated that different acupoints perform different hypoglycemic effects [26, 27].

According to traditional Chinese medicine (TCM), weiwanxiashu (EX-B3) is specialized in treatment of diabetes and is believed to have outstanding effect [28]. It is one of the most frequently selected ones in clinical and experimental studies of acupuncture treatment of T2DM reported in Chinese [29–31] and is proved to have outstanding effects on hyperglycemia and insulin resistance of T2DM, but little was reported in English [28, 32]. And the mechanism has not been well elucidated [28]. We hypothesized that weiwanxiashu (EX-B3) has better effects on hyperglycemia and insulin resistance of T2DM.

This study aims at observing EA weiwanxiashu's (EX-B3's) effects on hyperglycemia and insulin resistance of high fat diet combined with STZ-induced T2DM rats and exploring their relation with skeletal muscle GLUT4 protein expression.

## 2. Experiment Method

### 2.1. Experimental Animals

Experiment animals are 65 clean level male Sprague-Dawley rats (160 ± 5 g) purchased from S.P.F limited, Beijing (license: SCXK 2011-004). Animals were maintained in animal facility at Beijing University of Chinese Medicine with a controlled environment of 23°C, relative humidity 60%, and 12 h light/dark cycle. Rats were housed 6/cage, with free access to food and water. All the experiment procedures were performed in accordance with WHO's International Guiding Principles for Biomedical Research Involving Animals. And the study protocol was approved by the Joint Ethical Review Committee of Beijing University of Chinese Medicine (R-20131219-7).

### 2.2. Model Preparation

All the rats were fed with ordinary rodent chow for 1 week for adaptation. After that, 12 rats were selected randomly according to body weight as normal control group (fed with ordinary rodent chow). Other animals (53 rats) were prepared as T2DM model rats by high fat diet combined with STZ injection according to previous researches [33–35]: Rats were fed with high fat diet (consisting of 70% ordinary rodent chow, 10% sucrose, 10% lard oil, and 10% yolk powder. All the rat chow was purchased from Beijing HFK Bioscience Co., Ltd. (license: SCXK 2014-008)) for 50 days, overnight fasted for 10 hours (from 22:00 to 8:00), and then intraperitoneally injected with 2% streptozocin (STZ) solution (STZ dissolved in 0.1 mol/L citrate buffer solution with a pH of 4.3) at a dose of 30 mg/kg.

Random blood glucose (RBG) was measured 72 h after the injection, and oral glucose tolerance test (OGTT) was conducted 14 days after the injection. Animals with RBG > 16.7 mmol/L and OGTT's 2 h time-point blood glucose > 11.1 mmol/L were selected as successfully prepared T2DM models. Altogether 36 rats were included as model animals.

During the 14 days between STZ injection and model evaluation, rats except those of the normal control group were fed with high fat diet.

### 2.3. Grouping and EA Intervention

According to fasting blood glucose (FBG) measured 14 days after STZ injection, T2DM model rats were divided by random block design into 3 groups. They are T2DM model group (*n* = 12), EA weiwanxiashu (EX-B3) group (*n* = 12), and sham EA group (*n* = 12). The intervention began at the same day of grouping. And, since the day of grouping, all animals were fed with ordinary rodent chow.

Rats in normal control group were nondiabetic normal rats. Rats in T2DM model group were T2DM model rats. Rats in both groups received no EA intervention. By comparing results between normal control group and T2DM model group, the study verifies the quality of T2DM model animals. By comparing results between T2DM model group and EA weiwanxiashu (EX-B3) group, the study observes EA weiwanxiashu's (EX-B3's) intervention effects on FBG and glucose tolerance and explores the mechanism of the effects. Sham EA group was set to clarify the genuine effects of EA, when compared with EA weiwanxiashu (EX-B3) group.

**Figure 1:**
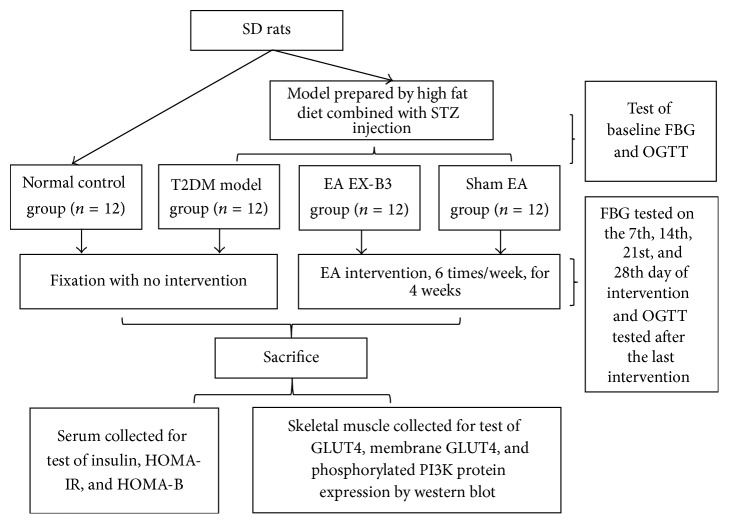
Experimental procedures.

*Details of Interventions*. The intervention was applied during 18:00–20:30, once every day (from Monday to Saturday), for 4 weeks, with a one-day interval between every 2 weeks. FBG was tested 8:00 on Sunday morning (i.e., the 7th, 14th, 21st, and 28th day of the intervention), with rats fasted overnight since 22:00, Saturday night (Figure 1).

*Details of EA Intervention*. The details of EA intervention are given as follows.

*Normal Control Group and T2DM Model Group*. Rats in 2 groups were fixed in loose white cotton bags, wait 5 min for adaptation, and stayed for 20 min, without any other intervention.

*EA Weiwanxiashu (EX-B3) Group*. Rats were fixed the same way as the normal control or T2DM model group, wait 5 minutes for adaptation. After that, needles of 0.16 mm × 7 mm (Beijing Zhongyan Taihe Medicine Co., Ltd, China. LOT: 031426) were inserted obliquely (towards the lateral side at an angle of 45 degrees) into weiwanxiashu (EX-B3) on both sides for 4 mm with help of a plastic tube to minimize the pain. When applying acupuncture, the needle was first put into a tube (which is 1.5 mm shorter than the needle) and pressed against skin of point area. By swiftly tapping on the needle handle outside the tube, the needle penetrated the skin for further adjustment of angle and depth. Soon after the insertion of needles, EA (2 Hz, 2 mA, continuous wave, 20 min) was applied on weiwanxiashu (EX-B3) by connecting the positive charge of EA device (KWD-808, Changzhou Yingdi Electronic Medical Device Co., Ltd., China) with needles on weiwanxiashu (EX-B3) and negative charge with a saline-infiltrated cotton ball pasted on rear foot of the same side. Weiwanxiashu (EX-B3) is located 7 mm lateral to the depression under the process of the 8th thoracic vertebrae.

*Sham EA Group*. Rats were fixed the same way as EA weiwanxiashu (EX-B3) group. After that, needles were inserted horizontally (towards the tip of tail at an angle of 15 degrees) into nonpoint (the mid-point of rats' tail on dorsal side) for 2 mm. Soon after the insertion, EA (2 mA, 2 Hz, continuous wave, 20 min) was applied by connecting nonpoint with a saline-infiltrated cotton ball pasted on the tip of the tail.

Fixation of animals, point locating, needle insertion, and EA device connection on all rats during the 4-week intervention were done by the same persons, respectively.

### 2.4. Detection of Indicators

#### 2.4.1. Measure of FBG

FBG was tested 1 day before the intervention and at the 7th, the 14th, the 21st, and the 28th day of the intervention using enzyme end-point method [36]. Blood sample was collected from the tail vein. Protocol: After disinfection of rat's tail, it was punctured with a blood taking needle (Tianjin huahong Technology Co., Ltd., Tianjin, China. LOT: 01-140804). After that, one drop of blood was collected on the test area of the test strip (Roche ACCU-CHEK ACTIVE test strip (ser. number GC12723032, LOT: 24626232)), with the test strip in the meter (Roche ACCU-CHEK ACTIVE blood glucose meter (ser. number GC12723032)). The reading was recorded as a result.

#### 2.4.2. Calculation of Area under Curve (AUC) of OGTT

OGTT was tested one day before the intervention and the day after the last intervention, to determine the effect of EA on rats' glucose tolerance. Protocol: All animals were fasted overnight from 22:00 to 8:00. After the test of FBG, rats were intragastrically given glucose solution at 2 g/kg body weight [37] and then tested for blood glucose 30 min, 60 min, and 120 min later. AUC of OGTT (mmol/L·h) was calculated. FBG was tested using enzyme end-point method mentioned above. Blood sample was collected from the tail vein [36].

#### 2.4.3. Test of Skeletal Muscle GLUT4 Protein Expression

After sacrifice of rats, same part of the quadriceps femoris of all rats was collected for western blot test of GLUT4 protein expression. Results were shown as the ratio to *β*-actin.

*Protocol of Western Blot Test*. For each group, 3 samples were selected randomly according to FBG tested before scarification.

For each sample, 100 mg of the tissue was ground in a 1.5 mL grinder with 1 mL radioimmunoprecipitation (RIPA) lysis buffer and protein inhibitor mixture in ice bath. The homogenate was then collected in a 1.5 mL centrifuge tube, placed on ice for 30 min (vortexed every 5 min), and then centrifuged (4°C, 10000 rpm, 15 min). The liquid supernatant was then collected for measurement of protein concentration with Bicinchoninic acid (BCA) kit, prepared at a balanced concentration of 4.67 *μ*g/*μ*L, and then denatured in 95°C water for 5 min.

For western blot test, polyacrylamide gel electrophoresis (80 V, 30 min for stacking gel, and 120 V, 60 min for separating gel) was made using 10% separating gel. The sample volume is 15 *μ*L–70 *μ*g. Proteins of different groups were wet-transferred (80 V, 70 min) to Polyvinylidene Fluoride (PVDF) microporous membrane (0.45 *μ*m in diameter), blocked 1 h in 5% w/v skimmed milk, 1x TBS, and 0.1% Tween 20 at 25°C with gentle shaking, and then incubated with 1 : 2000 diluted antibody (GLUT4 polyclonal antibody purchased from Abcam, LOT: GR183616-1) in 0.5% w/v skimmed milk, 1x TBS, and 0.1% Tween 20 at 25°C with gentle shaking for 1 h and at 4°C for 16 h. After that, the primary antibody was discarded, and the membrane was washed 3 times (5 min each time) with 1x TBST, incubated with 1 : 2000 diluted secondary antibody in 0.5% w/v skimmed milk, 1x TBS, and 0.1% Tween 20 at 25°C with gentle shaking for 1 h, and washed 3 times (5 min each time) with 1x TBST. The photo was taken in darkroom. And integrated optical density (IOD) was calculated with ImageProPlus 6.0.

#### 2.4.4. Test of Membrane GLUT4 Expression of Skeletal Muscle

After sacrifice of rats, same part of the quadriceps femoris of all rats was collected. Membrane proteins were extracted with Eukaryotic Membrane Protein Extraction Reagent Kit (Thermo Scientific, LOT: OJ186747A). Other procedures are the same as protocol of western blot test listed above. Results were shown as the ratio to *β*-actin.

#### 2.4.5. Observation of Translocation of GLUT4 to the Membrane of Skeletal Muscle

Comparative ratio of skeletal muscle membrane GLUT4 expression was shown as the ratio of skeletal muscle membrane GLUT4 expression to GLUT4 expression of the whole cell.

#### 2.4.6. Test of Phosphorylated Phosphatidylinositol-3-Kinase (PI3K) Expression of Skeletal Muscle

After sacrifice of rats, the same part of the quadriceps femoris of all rats was collected for western blot test of PI3K (Y607) protein expression. Protein were extracted with phosphatase inhibitor. Other procedures are the same as protocol of western blot test listed above (PI3K polyclonal antibody purchased from Abcam, LOT: 194710-8). Results were shown as the ratio to *β*-actin.

### 2.5. Statistical Treatment

SPSS 19.0 was applied for the processing of data. Results were shown as $\overset{-}{X}\pm s$. 0.01 < *P* < 0.05 was defined as significant difference and *P* < 0.01 as extremely significant difference. One-way analysis of variance (ANOVA) was applied for the comparison of serum insulin content and skeletal muscle cytoplasmic and membrane GLUT4 expression. For other indices, multiple-group nonparametric test was applied due to variance nonhomogeneity.

## 3. Results

### 3.1. EA's Intervention Effect on FBG

Results show that, before intervention, FBG of T2DM model group, EA weiwanxiashu (EX-B3) group, and sham EA group are significantly higher than that of normal control group (*P* < 0.01), and no statistical difference can be observed between the 3 groups. After 4 weeks' intervention, FBG of T2DM model group, EA weiwanxiashu (EX-B3) group, and sham EA group is still remarkably higher than that of the normal control group (*P* < 0.01). EA weiwanxiashu (EX-B3) can greatly reduce T2DM model rats' FBG (*P* < 0.01). But no significant difference can be observed between EA weiwanxiashu (EX-B3) group and sham EA group (see Table 1).

### 3.2. EA's Intervention Effect on Glucose Tolerance

Results show that, before intervention, AUC of OGTT of T2DM model group, EA weiwanxiashu (EX-B3) group, and sham EA group are significantly higher than that of normal control group (*P* < 0.01). And no statistical difference can be observed between the 3 groups. After intervention, AUC of OGTT of T2DM model group, EA weiwanxiashu (EX-B3) group, and sham EA group are still remarkably higher than that of the normal control group (*P* < 0.01). EA weiwanxiashu (EX-B3) group's result is greatly lower than T2DM model group (*P* < 0.01). No statistical difference can be observed between EA weiwanxiashu (EX-B3) group and sham EA group (see Table 2).

### 3.3. EA's Intervention Effect on HOMA-IR and HOMA-B

Results show that, after intervention, HOMA-IR of T2DM model group, EA weiwanxiashu (EX-B3) group, and sham EA group is greatly higher than that of the normal control group (*P* < 0.01). EA weiwanxiashu (EX-B3) can reduce rats' HOMA-IR to an extent significantly lower than T2DM model group (*P* < 0.01). HOMA-B of T2DM model group, EA weiwanxiashu (EX-B3) group, and sham EA group is greatly lower than that of normal control group (*P* < 0.01). EA weiwanxiashu (EX-B3) group can significantly increase the reading, compared with T2DM model group (*P* < 0.05) and sham EA group (*P* < 0.05) (see Table 3).

### 3.4. EA's Intervention Effect on Skeletal Muscle GLUT4 Protein Expression

Results show that T2DM model animals are of remarkably lowered GLUT4 protein expression in skeletal muscle (*P* < 0.01). Neither EA weiwanxiashu (EX-B3) nor sham EA can effectively upregulate GLUT4 expression in skeletal muscle (see Table 4).

Membrane GLUT4 expression of the normal control group is significantly higher than T2DM model group, EA weiwanxiashu (EX-B3) group, and sham EA group (*P* < 0.01). EA weiwanxiashu (EX-B3) can greatly improve GLUT4 protein expression in the membrane, compared with T2DM model group (*P* < 0.01) and sham EA group (*P* < 0.05) (see Table 4).

Comparative ratio of skeletal muscle membrane GLUT4 of T2DM model animals is greatly lower than normal control group (*P* < 0.01). EA weiwanxiashu (EX-B3) can effectively upregulate the ratio of GLUT4 in membrane of skeletal muscle, compared with T2DM model group and sham EA group (*P* < 0.01, *P* < 0.05), indicating stimulation of membrane translocation of GLUT4. However, the reading is still greatly lower than that of the normal control group (*P* < 0.01) (see Table 4).

Skeletal muscle PI3K (Y607) protein expression of T2DM model group is significantly lower than that of the normal control group (*P* < 0.05). EA weiwanxiashu (EX-B3) can greatly improve PI3K (Y607) expression in skeletal muscle (*P* < 0.01). Besides, its expression is remarkably higher than sham EA group (*P* < 0.01) (see Table 5).

## 4. Discussion

According to TCM, back-*shu* point is where* qi* of its corresponding* zang-fu* organs gathers. Back-*shu* point is therefore especially useful for treatment of diseases of its related* zang-fu* organ [38]. Weiwanxiashu (EX-B3) is the back-*shu* point of the pancreas and is therefore believed to be useful for treatment of T2DM. The point was frequently selected in clinical trial or animal experiment studies of T2DM in China, and many reports published in Chinese have proved its outstanding effect [29–31]. However, only a little can be searched [28, 32] due to language barrier, and the mechanism of weiwanxiashu (EX-B3) remains to be elucidated.

**Figure 2:**
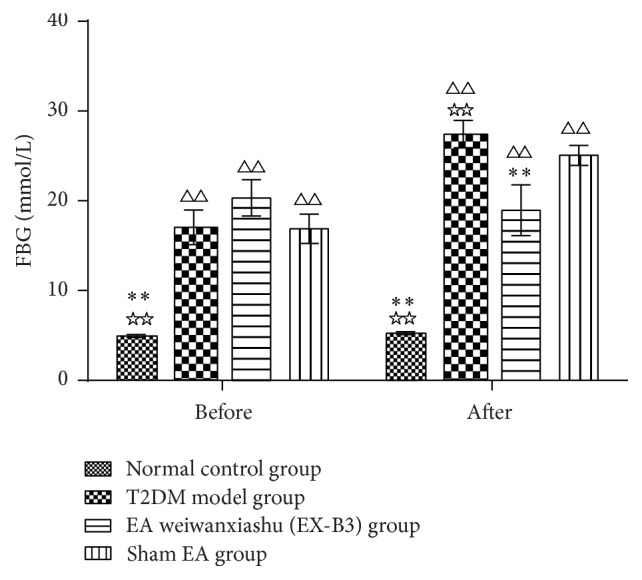
EA's intervention effect on FBG. Note: △△ for *P* < 0.01, versus normal control group. *∗∗* for *P* < 0.01, versus T2DM model group. ☆☆ for *P* < 0.01, versus EA weiwanxiashu (EX-B3) group.

**Figure 3:**
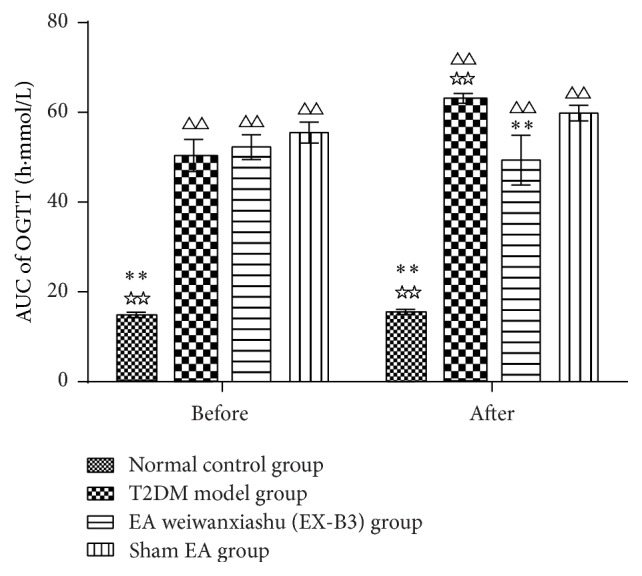
EA's intervention effect on AUC of OGTT. Note: △△ for *P* < 0.01, versus normal control group. *∗∗* for *P* < 0.01, versus T2DM model group. ☆☆ for *P* < 0.01, versus EA weiwanxiashu (EX-B3) group.

**Figure 4:**
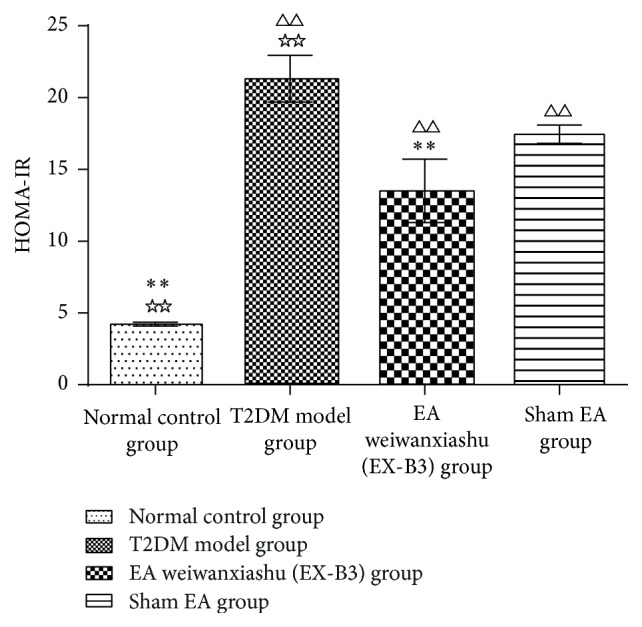
EA's intervention effect on HOMA-IR. Note: △△ for *P* < 0.01, versus normal control group. *∗∗* for *P* < 0.01, versus T2DM model group. ☆☆ for *P* < 0.01, versus EA weiwanxiashu (EX-B3) group.

**Figure 5:**
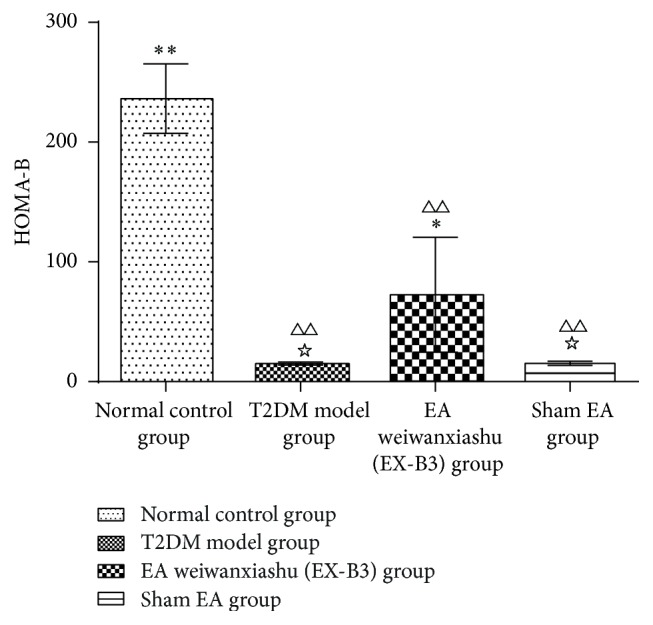
EA's intervention effect on HOMA-B. Note: △△ for *P* < 0.01, versus normal control group. *∗* for 0.01 < *P* < 0.05, versus T2DM model group, and *∗∗* for *P* < 0.01. ☆ for 0.01 < *P* < 0.05, versus EA weiwanxiashu (EX-B3) group.

Results of our study proved that EA weiwanxiashu (EX-B3) can significantly reduce model rats' highly rocketed FBG (Table 1, Figure 2), AUC of OGTT (Table 2, Figure 3), and HOMA-IR (Table 3, Figure 4) and greatly increase model rats' remarkably decreased HOMA-B (Table 3, Figure 5).

The above changes can be performed by activation of GLUT4. In static state, GLUT4 is sequestered in intracellular vesicles in skeletal muscle cells and has no biological activity. When stimulated by insulin signal or muscle contraction [39], vesicles that contain GLUT4 will fuse with the plasma membrane; GLUT4 was then inserted in the membrane and becomes available for active transporting glucose into the skeletal muscle cell [40]. As a result, blood glucose will be reduced.

Previous researches show that it is possible to regulate GLUT4 expression by EA [41–46]. It is proved that EA can normalize insulin sensitivity of polycystic ovary syndrome rats by increasing skeletal muscle cytoplasmic GLUT4 content [41]. The effect can also be observed in prednisolone induced insulin resistance rats: EA can greatly increase model rats significantly decreased cytoplasmic GLUT4 expression to restore free fatty acid content and HOMA-IR to normal [42]. Some researchers point out that EA cannot modify skeletal muscle cytoplasmic GLUT4 content. Instead, it mediates glucose uptake through translocation of GLUT4 from cytoplasm to membrane in response to activation of adenosine monophosphate-activated protein kinase (AMPK) or insulin signal pathways [43, 44]. Tominaga and colleagues show that EA can significantly improve glucose infusion in hyperinsulinaemic-euglycaemic clamp test by upregulating membrane but not cytoplasmic GLUT4 expression in skeletal muscle of insulin resistance rats prepared by high fructose diet [45]. The effect was also observed in adipose tissue [45]. Van Epps-Fung and colleagues show that translocation of GLUT4 from the cytosol to membrane improves glucose uptake in adipose tissue of insulin resistance rats induced by glucocorticoids [46]. To sum up, EA upregulates cytoplasmic GLUT4 expression or translocation of GLUT4 to improve skeletal muscle glucose uptake and relieve insulin resistance.

Our research results show that EA weiwanxiashu (EX-B3) can significantly improve hyperglycemia and insulin resistance of model rats and greatly improve skeletal muscle membrane GLUT4 expression but not cytoplasmic GLUT4 expression, indicating that EA can stimulate GLUT4 membrane translocation to reduce FBG and relieve insulin resistance. Test of skeletal muscle PI3K (Y607) protein expression demonstrates that, in our study, upregulation of GLUT4 membrane translocation is related to phosphorylation of PI3K.

This is in accordance with previous research conclusion that insulin signal takes effects in the skeletal muscle by binding insulin with the alpha-subunits of insulin receptor on the surface of cell. After that, beta-subunit of insulin receptor will autophosphorylate and lead to activating tyrosine phosphorylation of insulin receptor substrate (IRS; in skeletal muscle, it is mainly IRS-1) [47]. Phosphorylated IRS-1 will bind with regulatory subunit of phosphatidylinositol 3-kinase (PI3K) with Src homology 2 domain and further activate PI3K and protein kinase B (Akt). As a result, GLUT4 will translocate from the cytosol to the membrane and helps with active transport of glucose into the skeletal muscle [48–51].

**Figure 6:**
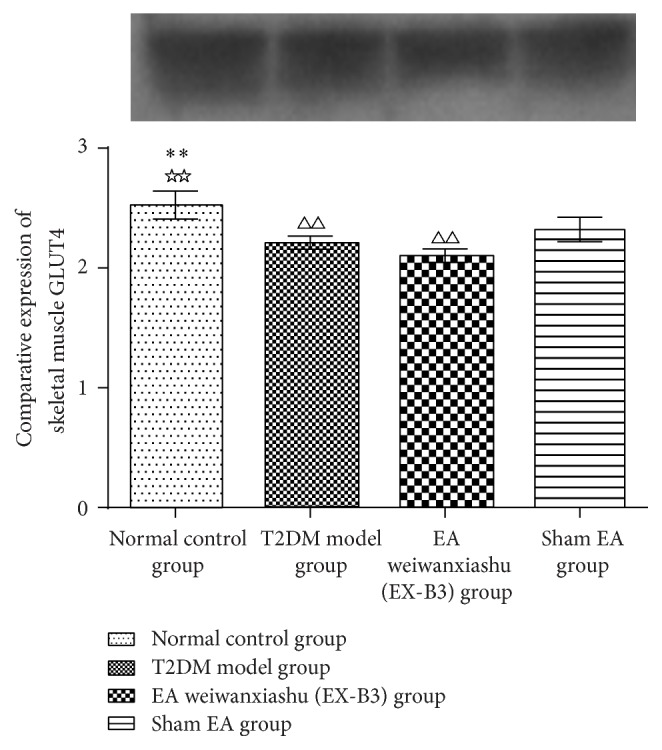
EA's intervention effect on skeletal muscle GLUT4 protein expression. Note: △△ for *P* < 0.01, versus normal control group. *∗∗* for *P* < 0.01, versus T2DM model group. ☆☆ for *P* < 0.01, versus EA weiwanxiashu (EX-B3) group.

**Figure 7 fig7:**
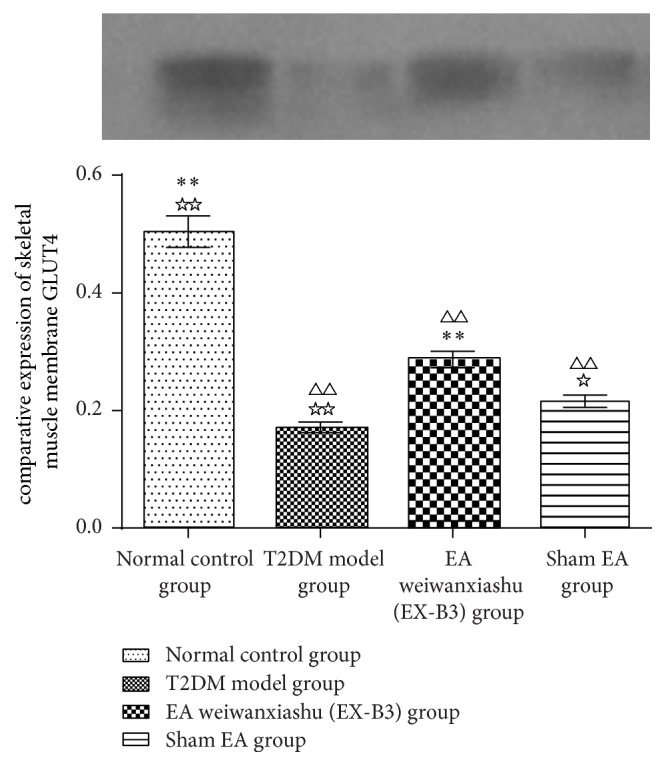
EA's intervention effect on skeletal muscle membrane GLUT4 protein expression. Note: △△ for *P* < 0.01, versus normal control group. *∗∗* for *P* < 0.01, versus T2DM model group. ☆ for 0.01 < *P* < 0.05, versus EA weiwanxiashu (EX-B3) group, and ☆☆ for *P* < 0.01.

**Figure 8:**
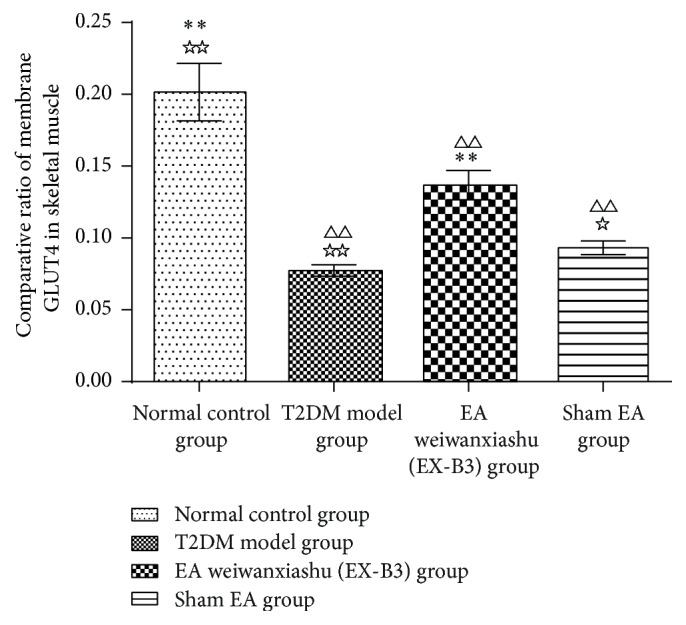
Comparative ratio of membrane GLUT4. Note: △△ for *P* < 0.01, versus normal control group. *∗∗* for *P* < 0.01, versus T2DM model group. ☆ for 0.01 < *P* < 0.05, versus EA weiwanxiashu (EX-B3) group, and ☆☆ for *P* < 0.01.

**Figure 9:**
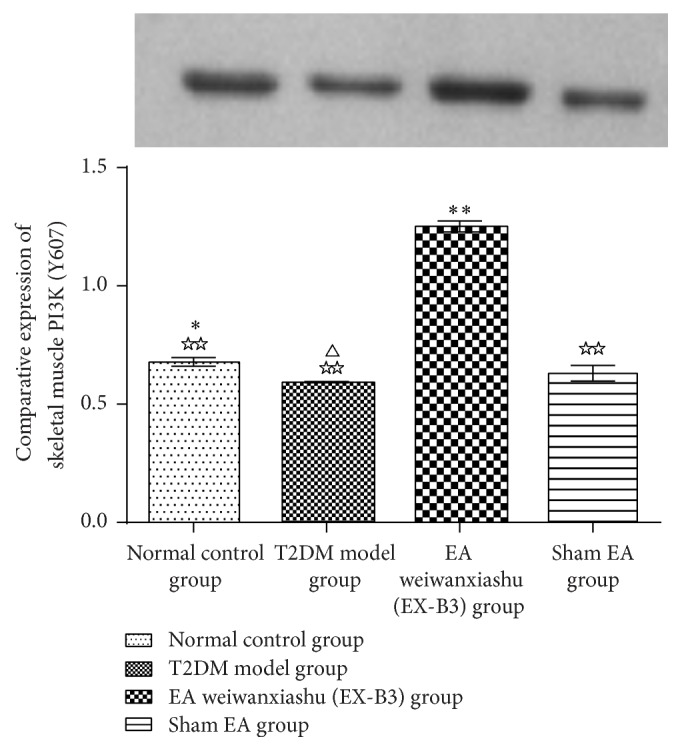
EA's intervention effect on skeletal muscle PI3K (Y607) protein expression. Note: △ for 0.01 < *P* < 0.05, versus normal control group. *∗* for 0.01 < *P* < 0.05, versus T2DM model group, and *∗∗* for *P* < 0.01. ☆☆ for *P* < 0.01, versus EA weiwanxiashu (EX-B3) group.

Since in our study, EA weiwanxiashu (EX-B3) group exceeds sham EA group in regulation of skeletal phosphorylated PI3K expression (Table 5, Figure 9), membrane translocation of GLUT4 (Table 4, Figures 6 [Fig fig7]–8), and HOMA-B (Table 3, Figure 5) and has better effect on FBG (Table 1, Figure 2), AUC of OGTT (Table 2, Figure 3), and HOMA-IR (Table 3, Figure 4). It rules out the possibility that EA weiwanxiashu's (EX-B3's) effects are done by placebo effect of EA.

EA weiwanxiashu (EX-B3) is especially useful for intervention of T2DM.

## 5. Conclusion

EA weiwanxiashu (EX-B3) can upregulate skeletal muscle phosphorylated PI3K protein expression, to stimulate membrane translocation of GLUT4 and thereby increase skeletal muscle glucose intake to reduce blood glucose and relieve insulin resistance.

## Figures and Tables

**Table 1:** EA's intervention effect on FBG ($\overset{-}{X}\pm s$, mmol/L).

Groups	*n*	FBG before intervention	FBG at the 7th day	FBG at the 14th day	FBG at the 21st day	FBG after intervention
Normal control group	12	4.95 ± 0.16^*∗∗*☆☆^	4.29 ± 0.47^*∗∗*☆☆^	4.93 ± 0.16^*∗∗*☆☆^	6.16 ± 0.18^*∗∗*☆☆^	5.24 ± 0.16^*∗∗*☆☆^
T2DM model group	9	17.04 ± 1.94^△△^	17.30 ± 3.22^△△^	15.88 ± 1.69^△△^	28.46 ± 1.04^△△☆☆^	27.42 ± 1.52^△△☆☆^
EA weiwanxiashu (EX-B3) group	10	20.33 ± 2.01^△△^	13.91 ± 2.15^△△^	18.38 ± 2.44^△△^	19.88 ± 2.92^△△*∗∗*^	18.95 ± 2.83^△△*∗∗*^
Sham EA group	9	16.88 ± 1.63^△△^	16.73 ± 2.26^△△^	17.63 ± 2.65^△△^	22.34 ± 2.24^△△*∗*^	25.07 ± 1.11^△△^

Note: △△ for *P *< 0.01, versus normal control group. *∗* for 0.01 < *P* < 0.05, versus T2DM model group, and *∗∗* for *P* < 0.01. ☆☆ for *P* < 0.01, versus EA weiwanxiashu (EX-B3) group.

**Table 2:** EA's intervention effect on AUC of OGTT ($\overset{-}{X}\pm s$, mmol/L·h).

Group	*n*	AUC of OGTT before intervention	AUC of OGTT after intervention
Normal control group	12	14.89 ± 0.62^*∗∗*☆☆^	15.55 ± 0.57^*∗∗*☆☆^
T2DM model group	9	50.38 ± 3.55^△△^	63.14 ± 1.09^△△☆☆^
EA weiwanxiashu (EX-B3) group	10	52.27 ± 2.76^△△^	49.37 ± 5.52^△△*∗∗*^
Sham EA group	9	55.50 ± 2.33^△△^	59.84 ± 1.76^△△^

Note: △△ for *P *< 0.01, versus normal control group. *∗∗* for *P* < 0.01, versus T2DM model group. ☆☆ for *P* < 0.01, versus EA weiwanxiashu (EX-B3) group.

**Table 3:** EA's intervention effect on HOMA-IR and HOMA-B ($\overset{-}{X}\pm s$).

Group	*n*	Fasting insulin (mmol/L)	HOMA-IR	HOMA-B
Normal control group	12	18.24 ± 0.65^☆^	4.22 ± 0.14^*∗∗*☆☆^	236.41 ± 28.96^*∗∗*^
T2DM model group	9	17.48 ± 0.81	21.32 ± 1.62^△△☆☆^	15.08 ± 1.18^△△☆^
EA weiwanxiashu (EX-B3) group	10	16.01 ± 0.80^△^	13.51 ± 2.20^△△*∗∗*^	72.65 ± 48.07^△△*∗*^
Sham EA group	9	15.94 ± 1.00^△^	17.46 ± 0.63^△△^	15.39 ± 1.68^△△☆^

Note: △ for 0.01 < *P *< 0.05, versus normal control group, and △△ for *P *< 0.01. *∗* for 0.01 < *P *< 0.05, versus T2DM model group, and *∗∗* for *P *< 0.01. ☆ for 0.01 < *P *< 0.05, versus EA weiwanxiashu (EX-B3) group, and ☆☆ for *P *< 0.01. HOMA-IR = FBG × fasting blood insulin/22.5. HOMA-B = fasting blood insulin × 20/(FBG − 3.5).

**Table 4:** EA's intervention effect on skeletal muscle GLUT4 protein expression ($\overset{-}{X}\pm s$).

Group	*n*	GLUT4	Membrane GLUT4	Comparative ratio of membrane GLUT4
Normal control group	3	2.53 ± 0.12^*∗∗*☆☆^	0.50 ± 0.03^*∗∗*☆☆^	0.20 ± 0.02^*∗∗*☆☆^
T2DM model group	3	2.21 ± 0.06^△△^	0.17 ± 0.01^△△☆☆^	0.08 ± 0.00^△△☆☆^
EA weiwanxiashu (EX-B3) group	3	2.10 ± 0.06^△△^	0.29 ± 0.01^△△*∗∗*^	0.14 ± 0.01^△△*∗∗*^
Sham EA group	3	2.32 ± 0.10	0.22 ± 0.01^△△☆^	0.09 ± 0.00^△△☆^

Note: △△ for *P *< 0.01, versus normal control group. *∗∗* for *P *< 0.01, versus T2DM model group. ☆ for 0.01 < *P *< 0.05, versus EA weiwanxiashu (EX-B3) group, and ☆☆ for *P *< 0.01.

**Table 5:** EA's intervention effect on skeletal muscle PI3K (Y607) protein expression ($\overset{-}{X}\pm s$).

Group	*n*	PI3K (Y607)
Normal control group	3	0.68 ± 0.02^*∗*☆☆^
T2DM model control group	3	0.59 ± 0.00^△☆☆^
EA weiwanxiashu (EX-B3) group	3	1.25 ± 0.02^*∗∗*^
Sham EA group	3	0.63 ± 0.03^☆☆^

Note: △ for 0.01 < *P *< 0.05, versus normal control group. *∗* for 0.01 < *P *< 0.05, versus T2DM model group, and *∗∗* for *P *< 0.01. ☆☆ for *P *< 0.01, versus EA weiwanxiashu (EX-B3) group.

## References

[1] Guariguata L. (2012). By the numbers: new estimates from the IDF Diabetes Atlas Update for 2012. *Diabetes Research and Clinical Practice*.

[2] Whiting D. R., Guariguata L., Weil C., Shaw J. (2011). IDF diabetes atlas: global estimates of the prevalence of diabetes for 2011 and 2030. *Diabetes Research and Clinical Practice*.

[3] Lorenz M., Evers A., Wagner M. (2013). Recent progress and future options in the development of GLP-1 receptor agonists for the treatment of diabesity. *Bioorganic and Medicinal Chemistry Letters*.

[4] Taylor R. (2012). Insulin resistance and type 2 diabetes. *Diabetes*.

[5] Mehra B. R., Thawait A. P., Karandikar S. S., Gupta D. O., Narang R. R. (2008). Evaluation of foot problems among diabetics in rural population. *Indian Journal of Surgery*.

[6] Levelt E., Mahmod M., Piechnik S. K. (2016). Relationship between left ventricular structural and metabolic remodeling in type 2 diabetes. *Diabetes*.

[7] Danaei G., Finucane M. M., Lu Y. (2011). National, regional, and global trends in fasting plasma glucose and diabetes prevalence since 1980: systematic analysis of health examination surveys and epidemiological studies with 370 country-years and 2·7 million participants. *The Lancet*.

[8] Chen L.-N., Lyu J., Yang X.-F. (2013). Liraglutide ameliorates glycometabolism and insulin resistance through the upregulation of GLUT4 in diabetic KKAy mice. *International Journal of Molecular Medicine*.

[9] Rea S., James D. E. (1997). Moving GLUT4: the biogenesis and trafficking of GLUT4 storage vesicles. *Diabetes*.

[10] Thurmond D. C., Pessin J. E. (2000). Discrimination of GLUT4 vesicle trafficking from fusion using a temperature-sensitive Munc18c mutant. *The EMBO Journal*.

[11] Tan Z., Zhou L.-J., Mu P.-W. (2012). Caveolin-3 is involved in the protection of resveratrol against high-fat-diet-induced insulin resistance by promoting GLUT4 translocation to the plasma membrane in skeletal muscle of ovariectomized rats. *Journal of Nutritional Biochemistry*.

[12] Cunha V. N., de Paula Lima M., Motta-Santos D. (2015). Role of exercise intensity on GLUT4 content, aerobic fitness and fasting plasma glucose in type 2 diabetic mice. *Cell Biochemistry & Function*.

[13] Fang P., Yu M., He B. (2016). Central injection of GALR1 agonist M617 attenuates diabetic rat skeletal muscle insulin resistance through the Akt/AS160/GLUT4 pathway. *Mechanisms of Ageing and Development*.

[14] Lee J. O., Song Y.-H., Kim M.-W. (2013). A sub-1-volt nanoelectromechanical switching device. *Nature Nanotechnology*.

[15] Zisman A., Peroni O. D., Abel E. D. (2000). Targeted disruption of the glucose transporter 4 selectively in muscle causes insulin resistance and glucose intolerance. *Nature Medicine*.

[16] Hansen P. A., Gulve E. A., Marshall B. A. (1995). Skeletal muscle glucose transport and metabolism are enhanced in transgenic mice overexpressing the Glut4 glucose transporter. *The Journal of Biological Chemistry*.

[17] Giannocco G., Oliveira K. C., Crajoinas R. O. (2013). Dipeptidyl peptidase IV inhibition upregulates GLUT4 translocation and expression in heart and skeletal muscle of spontaneously hypertensive rats. *European Journal of Pharmacology*.

[18] Fu Y., Luo L., Luo N., Zhu X., Garvey W. T. (2007). NR4A orphan nuclear receptors modulate insulin action and the glucose transport system: potential role in insulin resistance. *The Journal of Biological Chemistry*.

[19] Brozinick J. T., McCoid S. C., Reynolds T. H. (2001). GLUT4 overexpression in db/db mice dose-dependently ameliorates diabetes but is not a lifelong cure. *Diabetes*.

[20] Magnoni L. J., Vraskou Y., Palstra A. P., Planas J. V. (2012). AMP-activated protein kinase plays an important evolutionary conserved role in the regulation of glucose metabolism in fish skeletal muscle cells. *PLoS ONE*.

[21] Nieto-Vazquez I., Fernández-Veledo S., De Alvaro C., Lorenzo M. (2008). Dual role of interleukin-6 in regulating insulin sensitivity in murine skeletal muscle. *Diabetes*.

[22] World Health Organization (2002). *Acupuncture: Review and Analysis of Reports on Controlled Clinical Trials*.

[23] Peplow P. V., Han S. M. (2014). Repeated application of electroacupuncture ameliorates hyperglycemia in obese Zucker diabetic fatty rats. *Journal of Acupuncture and Meridian Studies*.

[24] Ishizaki N., Okushi N., Yano T., Yamamura Y. (2009). Improvement in glucose tolerance as a result of enhanced insulin sensitivity during electroacupuncture in spontaneously diabetic Goto-Kakizaki rats. *Metabolism: Clinical and Experimental*.

[25] Chang S.-L., Lin K.-J., Lin R.-T., Hung P.-H., Lin J.-G., Cheng J.-T. (2006). Enhanced insulin sensitivity using electroacupuncture on bilateral Zusanli acupoints (ST 36) in rats. *Life Sciences*.

[26] Chang S. L., Lin J. G., Heish C. L. (2002). Comparison of hypoglycemic effect in different acupoint response to 2 Hz electroacupuncture. *Journal of Traditional Chinese Medicine*.

[27] Lin R.-T., Tzeng C.-Y., Lee Y.-C. (2014). Acupoint-specific, frequency-dependent, and improved insulin sensitivity hypoglycemic effect of electroacupuncture applied to drug-combined therapy studied by a randomized control clinical trial. *Evidence-Based Complementary and Alternative Medicine*.

[28] Zeng Z., Li Y. (2002). Effects of electroacupuncture at Weiwanxiashu and Zusanli points on blood glucose and plasma pancreatic glucagon contents in diabetic rabbits. *Journal of Traditional Chinese Medicine*.

[29] Liao H., Xi P., Chen Q., Yi L., Zhao Y. (2007). Clinical study on acupuncture, moxibustion, acupuncture plus moxibustion at Weiwanxiashu (EX-B3) for treatment of diabetes. *Zhongguo Zhen Jiu*.

[30] Wang Y.-D., Liu Z.-C., Xu B. (2014). Efficacy observation of acupuncture and tapping therapy in the treatment of type 2 diabetes of yin deficiency pattern combined with stasis in the patients. *Zhongguo Zhen Jiu*.

[31] Gao S., Li R., Tian H.-H., Pei E.-S., Cao B.-Y., Wu Y. (2014). Effects of electroacupuncture at ‘Yishu’ (EX-B 3) on the relative hormones of HPA axis in rats with type-2 diabetes mellitus. *Zhongguo Zhen Jiu*.

[32] Wu Y., Fei M., He Y. (2006). Clinical observation on senile patients with impaired glucose tolerance treated by point application. *Journal of Traditional Chinese Medicine*.

[33] Ji J., Zhang C., Luo X. (2015). Effect of stay-green wheat, a novel variety of wheat in China, on glucose and lipid metabolism in high-fat diet induced type 2 diabetic rats. *Nutrients*.

[34] Liu X.-Y., Liu F.-C., Deng C.-Y. (2016). Left ventricular deformation associated with cardiomyocyte Ca^2+^ transients delay in early stage of low-dose of STZ and high-fat diet induced type 2 diabetic rats. *BMC Cardiovascular Disorders*.

[35] Zheng X.-K., Zhang L., Wang W.-W., Wu Y.-Y., Zhang Q.-B., Feng W.-S. (2011). Anti-diabetic activity and potential mechanism of total flavonoids of *Selaginella tamariscina* (Beauv.) Spring in rats induced by high fat diet and low dose STZ. *Journal of Ethnopharmacology*.

[36] Zhang Q., Xiao X.-H., Li M. (2014). Chromium-containing traditional Chinese medicine, Tianmai Xiaoke tablet improves blood glucose through activating insulin-signaling pathway and inhibiting PTP1B and PCK2 in diabetic rats. *Journal of Integrative Medicine*.

[37] Oguma T., Kuriyama C., Nakayama K. (2015). The effect of combined treatment with canagliflozin and teneligliptin on glucose intolerance in Zucker diabetic fatty rats. *Journal of Pharmacological Sciences*.

[38] Cabioglu M. T., Arslan G. (2008). Neurophysiologic basis of Back-Shu and Huatuo-Jiaji points. *American Journal of Chinese Medicine*.

[39] Lund S., Holman G. D., Schmitz O., Pedersen O. (1995). Contraction stimulates translocation of glucose transporter GLUT4 in skeletal muscle through a mechanism distinct from that of insulin. *Proceedings of the National Academy of Sciences of the United States of America*.

[40] Cushman S. W., Wardzala L. J. (1980). Potential mechanism of insulin action on glucose transport in the isolated rat adipose cell. Apparent translocation of intracellular transport systems to the plasma membrane. *The Journal of Biological Chemistry*.

[41] Johansson J., Feng Y., Shao R., Lönn M., Billig H., Stener-Victorin E. (2010). Intense electroacupuncture normalizes insulin sensitivity, increases muscle GLUT4 content, and improves lipid profile in a rat model of polycystic ovary syndrome. *American Journal of Physiology—Endocrinology and Metabolism*.

[42] Lin R.-T., Tzeng C.-Y., Lee Y.-C. (2009). Acute effect of electroacupuncture at the Zusanli acupoints on decreasing insulin resistance as shown by lowering plasma free fatty acid levels in steroid-background male rats. *BMC Complementary and Alternative Medicine*.

[43] Hayashi T., Hirshman M. F., Kurth E. J. (1998). Evidence for 5′ AMP-activated protein kinase mediation of the effect of muscle contraction on glucose transport. *Diabetes*.

[44] Shepherd P. R., Kahn B. B. (1999). Glucose transporters and insulin action: implications for insulin resistance and diabetes mellitus. *The New England Journal of Medicine*.

[45] Tominaga A., Ishizaki N., Naruse Y., Kitakoji H., Yamamura Y. (2011). Repeated application of low-frequency electroacupuncture improves high-fructose diet-induced insulin resistance in rats. *Acupuncture in Medicine*.

[46] Van Epps-Fung M., Williford J., Wells A., Hardy R. W. (1997). Fatty acid-induced insulin resistance in adipocytes. *Endocrinology*.

[47] Garrido P., Morán J., Alonso A., González S., González González C. (2013). 17*β*-estradiol activates glucose uptake via GLUT4 translocation and PI3K/Akt signaling pathway in MCF-7 Cells. *Endocrinology*.

[48] Gandhi G. R., Jothi G., Antony P. J. (2014). Gallic acid attenuates high-fat diet fed-streptozotocin-induced insulin resistance via partial agonism of PPAR*γ* in experimental type 2 diabetic rats and enhances glucose uptake through translocation and activation of GLUT4 in PI3K/p-Akt signaling pathway. *European Journal of Pharmacology*.

[49] Ramachandran V., Saravanan R. (2015). Glucose uptake through translocation and activation of GLUT4 in PI3K/Akt signaling pathway by asiatic acid in diabetic rats. *Human and Experimental Toxicology*.

[50] Nagano K., Takeuchi H., Gao J. (2015). Tomosyn is a novel Akt substrate mediating insulin-dependent GLUT4 exocytosis. *International Journal of Biochemistry and Cell Biology*.

[51] Gandhi G. R., Stalin A., Balakrishna K., Ignacimuthu S., Paulraj M. G., Vishal R. (2013). Insulin sensitization via partial agonism of PPAR*γ* and glucose uptake through translocation and activation of GLUT4 in PI3K/p-Akt signaling pathway by embelin in type 2 diabetic rats. *Biochimica et Biophysica Acta—General Subjects*.

